# Assessing the effect of current steering on the total electrical energy delivered and ambulation in Parkinson’s disease

**DOI:** 10.1038/s41598-020-64250-7

**Published:** 2020-05-19

**Authors:** Daphne Hui, Aditya A. Murgai, Greydon Gilmore, Shabna I. Mohideen, Andrew G. Parrent, Mandar S. Jog

**Affiliations:** 1 Department of Physiology and Pharmacology, Western University, London, ON N6A 5C1 Canada; 2 Department of Clinical Neurological Sciences, Western University, London, ON N6A 3K7 Canada; 3 School of Biomedical Engineering, Western University, London, ON N6A 3K7 Canada

**Keywords:** Neurosurgery, Parkinson's disease, Parkinson's disease, Translational research, Parkinson's disease

## Abstract

Vertical current steering (vCS) divides current between multiple contacts, which reduces radial spread to fine-tune the electric field shape and improves neuroanatomical targeting. vCS may improve the variable responsiveness of Parkinsonian gait to conventional deep brain stimulation. We hypothesized that vCS elicits greater improvement in ambulation in Parkinson’s disease patients compared to conventional, single-contact stimulation. vCS was implemented with divisions of 70%/30% and 50%/50% and compared to single-contact stimulation with four therapeutic window amplitudes in current-controlled systems. Walking at a self-selected pace was evaluated in seven levodopa-responsive patients. Integrative measures of gait and stimulation parameters were assessed with the functional ambulation performance (FAP) score and total electrical energy delivered (TEED), respectively. A two-tailed Wilcoxon matched-pairs signed rank test assessed the effect of each stimulation condition on FAP and TEED and compared regression slopes; further, a two-tailed Spearman test identified correlations. vCS significantly lowered the TEED (*P* < 0.0001); however, FAP scores were not different between conditions (*P* = 0.786). Compared to single-contact stimulation, vCS elicited higher FAP scores with lower TEED (*P* = 0.031). FAP and TEED were positively correlated in vCS (*P* = 2.000 × 10^-5^, r = 0.397) and single-contact stimulation (*P* = 0.034, r = 0.205). Therefore, vCS and single-contact stimulation improved ambulation similarly but vCS reduced the TEED and side-effects at higher amplitudes.

## Introduction

## Parkinson’s disease and deep brain stimulation

Deep brain stimulation (DBS) consistently relieves appendicular symptoms in Parkinson’s disease (PD) but its effect on Parkinsonian gait deficits remains more elusive and variable^1^. Thus, in present clinical practice, PD patients with severe postural and gait instabilities or levodopa (L-DOPA) resistant postural and gait instabilities are often excluded from receiving DBS^2–4^. DBS chronically transmits electrical pulses from a sub-dermally implanted impulse generator (IPG) in the upper chest to neural tissue through implanted electrodes to address disabling dyskinesia and motor fluctuations from long-term use of L-DOPA^5^. The varying response of Parkinsonian gait deficits to DBS may be attributed to extensive physiological integration of various networks necessary for gait facilitation, the influence of bradykinesia and rigidity on Parkinsonian gait deficits, and the bias of upper limb symptom improvement during contact localization and selection^6,7^. Gait deficits attributed to rigidity and bradykinesia such as reduced step length and gait velocity tend to respond to L-DOPA and subthalamic nucleus (STN)-DBS. However, L-DOPA responsive gait deficits still exhibit a more variable responsiveness to STN-DBS as compared to appendicular symptoms such as tremor and rigidity. For instance, stimulation spread into the zona incerta and/or fields of forel from STN targeted electrodes has been reported to induce gait akinesia with a paradoxical improvement of dyskinesia, rigidity, and tremor^8,9^. This outcome highlights the sensitivity of gait treatment to neuroanatomical targeting of the resultant stimulation.

Three major goals of DBS programming are to 1) optimize symptom alleviation, 2) reduce side effects, and 3) limit IPG power consumption to reduce the battery drainage rate. Faster drainage of the IPG battery results in an earlier need for replacement, which may occur as early as three to five years post-implantation in non-rechargeable systems^10^. IPG replacements are associated with a higher infection rate compared to the initial implantation and inadequate symptom alleviation is common following replacements despite programming with previously successful stimulation parameters (e.g., frequency, pulse width, and current/voltage magnitude)^11,12^. The clinical outcome and power usage are predominantly influenced by the electrode geometry and stimulation parameter magnitudes^10^. Electrode geometry is determined by the designation of the cathode and anode; unipolar configurations assign the contact(s) and IPG as the cathode and anode, respectively, and bipolar configurations assign one contact and a different contact as the cathode and anode, respectively^10^. Conventionally, single-contact unipolar configurations are programmed first; followed by, double contact unipolar then bipolar configurations upon failure of the previous configuration^13^. Unipolar stimulation is associated with a lower therapeutic threshold; thus, symptom alleviation occurs at lower amplitudes, which is beneficial for reducing power consumption^10^. However, the generated electric field is relatively broad due to the large distance between the cathode and anode^10^. Current spreads radially and commonly induces side effects, which are more pronounced with poor localization but more likely at high amplitudes regardless of localization^10^. Bipolar stimulation is associated with a higher side effect threshold; thus, higher amplitudes may be administered before inducing side effects. Namely, bipolar and unipolar stimulation elicit similar clinical benefits at lower stimulation magnitudes but unipolar stimulation is associated with more side effects at higher magnitudes^10,14^. Accordingly, less current spread is associated with bipolar stimulation since charge distribution encompasses a smaller volume as the anode and cathode are relatively closer^10^. However, modelling studies demonstrated that bipolar configurations may be less selective than unipolar configurations because neural elements are activated at both the anode and cathode^15^.

Furthermore, an optimal combination of frequency, pulse width, and current/voltage magnitudes must address the clinical outcome and power consumption^10^. Lower magnitudes tend to reduce power consumption but may be insufficient for effective symptom alleviation. For instance, low frequency stimulation between 13-30 hertz (Hz) has been reported to worsen bradykinesia^16^. Overall, the current/voltage, frequency, pulse width, and impedance influencing the stimulation may be expressed as a single variable termed the total electrical energy delivered (TEED)^17,18^. TEED is directly related to the power consumption and IPG battery drainage rate; thus, it may allude to the practical efficacy of stimulation^19–22^. TEED was first introduced by Moro and colleagues; however, it was later modified by Koss and colleagues and reported as follows:

$${{\rm{TEED}}}_{1\sec }=\frac{{(voltage)}^{2}\times frequency\times pulse\,width}{impedance}\times 1\,second.$$

## Advances in DBS technology

Until recently, DBS systems have been pre-dominantly voltage-controlled (VC), which inconsistently deliver charge due to impedance fluctuations at the contiguous border between the neural tissue and contact^23,24^. Charge delivery of VC devices may not match the programmed value, which renders frequent programming adjustments due to inadequate stimulation of the target^13^. This is particularly an issue for programming during the early post-operative period (first few weeks following surgical implantation) when impedance fluctuations are common. Namely, oedema as a result of surgical implantation increases the electrode impedance while stimulation decreases impedance^25^. Accordingly, tedious programming prompted the development of current-controlled (CC) systems with multiple independent current control^13,23^. CC devices adjust voltage to account for impedance fluctuations to ensure that charge distribution is consistent and that current amplitudes match the programmed value^13,23^. Multiple independent current control allows for manipulation of current delivery from each contact as a separate entity, which allows for directional and vertical current steering (vCS)^26^. Directional current steering activates individual contact segments of the ring rather than stimulating in the ring-mode (across the entire electrode circumference) to fine-tune stimulation in the axial plane. Alternatively, vCS divides current between multiple contacts stimulating in the ring-mode along the dorsal-ventral axis of the electrode. vCS targets or avoids neural structures by shaping the stimulation field by manipulating the percentage of current that is distributed from an active contact, which is directly related to the electric field spread. Thus, contacts stimulating with a greater percentage will exhibit a broader spread. Accordingly, vCS serves as a systematic technique to fine-tune the stimulation field to improve the accuracy of neuroanatomical targeting and algorithms associating divisions to contact localizations may replace present, empirical approaches to DBS programming^13,27^.

## Rationale, objective, and hypothesis

vCS is a programming technique that may improve the clinical outcome by shaping the stimulation field. vCS reduces diffuse radial spread of the conventional spherical field emitted from a contact, which reduces side effects related to stimulation spread into non-intended neural structures. Additionally, systematically tailoring current divisions to contact localizations may improve the accuracy of targeting the array of interacting circuits that regulate gait^7^. Furthermore, employing separate current division programs may independently alleviate appendicular and axial symptoms to feasibly overcome the bias towards evaluating upper limb symptom improvement during contact localization and selection. Therefore, vCS may potentially address the variable improvement of L-DOPA responsive gait deficits feasibly and systematically.

A few studies have exhibited the effectiveness of utilizing vCS to treat PD; namely, the first case study found that vCS reduces the efficacy threshold and increases the side effect threshold^28^. Additionally, the first multicentre study reported that majority of participants initiated programming with single-contact stimulation but switched to vCS for optimal symptom improvement by the one year follow up^29^. Reduction of the efficacy threshold allows for symptom alleviation at lower amplitudes, which decreases power consumption and slows battery drainage. Overall, this suggests that vCS is an advantageous technique as it allows for feasible systematic programming, which may improve the clinical outcome more efficiently. Thus, the rationale was to assess the ability of vCS to fine-tune stimulation in order to minimize the ad-hoc programming conventionally used to improve L-DOPA responsive gait deficits. Namely, vCS may address impedance fluctuations during the early post-operative period and stimulation of un-intended neural structures that may elicit side effects or sub-optimal symptom alleviation. Aforementioned issues have been inconsistently addressed with unipolar and bipolar stimulation with conventional VC-DBS systems.

The purpose of our investigation was to compare the effect of vCS and single-contact stimulation on Parkinsonian gait deficits in CC devices. We hypothesized that vCS elicits a greater improvement in ambulation among PD patients compared to conventional, single-contact stimulation. The two main objectives were to 1) assess the individual effect of vCS and single-contact stimulation on integrative measures of gait parameters—functional ambulation performance (FAP) score and stimulation parameters—TEED, and to 2) determine the correlation and compare the relationship between FAP and TEED with vCS and single-contact stimulation.

## Results

**Figure 1:**
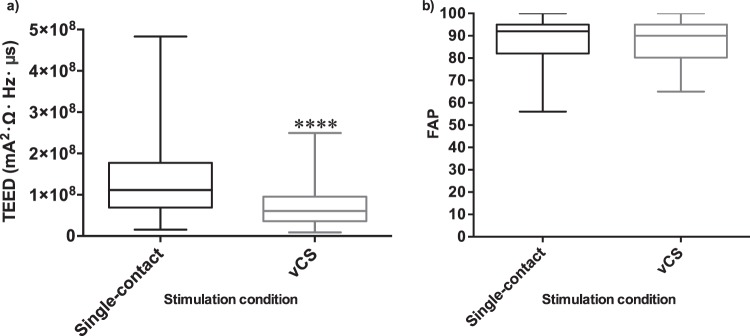
Effect of single-contact and vCS stimulation on TEED and FAP values. Means of TEED values and FAP scores compared in single-contact and vCS stimulation at all amplitude stages (around 20%, 40%, 60%, and 80% of the TW). Boxplots with N= 7; solid horizontal lines represent the median and the upper and lower whiskers represent the maximum and minimum values, respectively. (**a**) TEED values on average were significantly lower with vCS compared to single-contact stimulation (****, *P* < 0.0001; two-tailed, Wilcoxon matched-pairs signed rank test). (**b**) FAP scores on average were not different in the two stimulation conditions (*P* = 0.786; two-tailed, Wilcoxon matched-pairs signed rank test).

**Figure 2:**
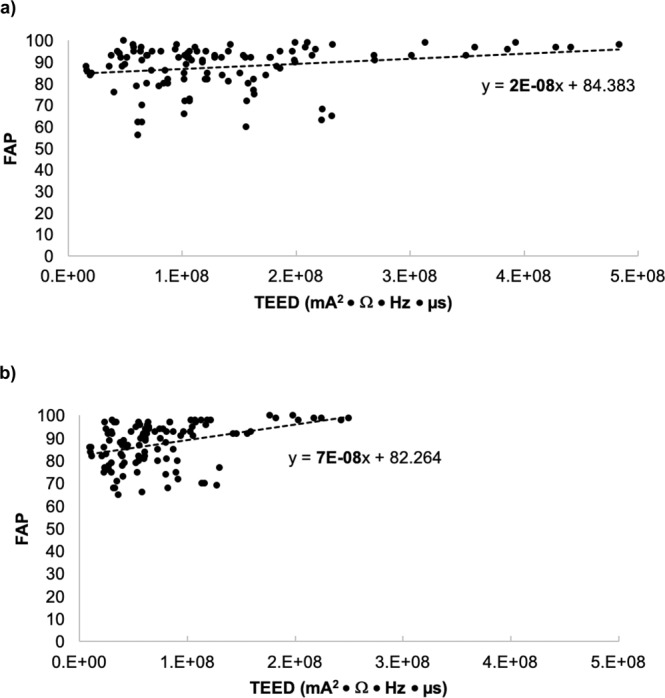
Correlation between TEED and FAP for single-contact and vCS stimulation. Correlation between TEED values and FAP scores during single-contact and vCS stimulation at all amplitude stages (around 20%, 40%, 60%, and 80% of the TW) with N= 7. (**a**) Positive correlation was found and the slope of the linear trend line was 2 × 10^-8^ with single-contact stimulation (*P* = 0.034, r = 0.205; two-tailed, Spearman correlation). (**b)** Positive correlation was found and the slope of the linear trend line was 7 × 10^-8^ with vCS stimulation (*P* = 2.000 × 10^-5^, r = 0.397; two-tailed, Spearman correlation).

**Figure 3:**
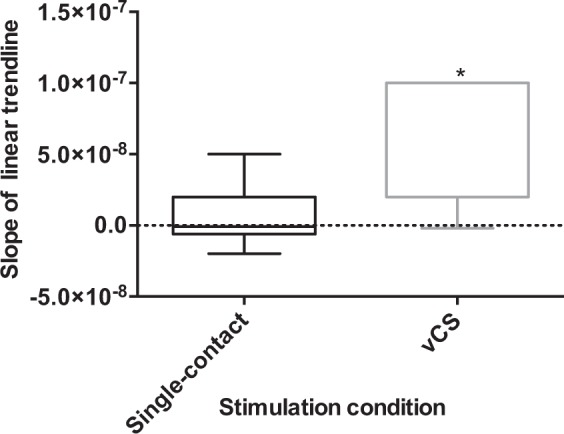
Comparison of the slope of the linear trend line of the regression between TEED and FAP in single-contact and vCS stimulation. Slope of the regression between TEED values and FAP scores with FAP set as the dependent variable during single-contact and vCS stimulation across all amplitude stages (around 20%, 40%, 60%, and 80% of the TW). Boxplots with N = 7; solid horizontal lines represent the median, and the upper and lower whiskers represent the maximum and minimum values, respectively. Slope of vCS stimulation was significantly greater than the slope of the single-contact stimulation condition (*P =0.031; two-tailed, Wilcoxon matched-pairs signed rank test).

The sample of seven participants exhibited an average disease duration and age of 12.5 and 65.3 ± 7.2 years, respectively; furthermore, 57.1% of the sample consisted of females. The average response to L-DOPA across all participants was 48.1% (Table 1). There were no differences between Unified Parkinson’s Disease Rating Scale (UPDRS) motor symptom scores of vCS and single-contact stimulation (*P* = 0.705) and across amplitude stages (*P* = 1.000) (Table 2). The average amplitude across all participants for Amplitude Stage 1, 2, 3, and 4 for the left STN was 1.8 milliamperes (mA), 2.4 mA, 2.9 mA, and 3.4 mA, respectively (Table 3). The average amplitude across all participants for Amplitude Stage 1, 2, 3, and 4 for the right STN was 1.7 mA, 2.3 mA, 3.0 mA, and 3.6 mA, respectively (Table 3). The TEED by vCS was significantly lower than single-contact stimulation (*P* < 0.0001) (Fig. 1a); however, the FAP score means of both conditions were not significantly different (*P* = 0.786) (Fig. 1b). A positive correlation was found between FAP and TEED with single-contact stimulation (r = 0.205, *P* = 0.034) (Fig. 2a) and vCS (r = 0.397, *P* = 2.000 × 10^-5^) (Fig. 2b). With the FAP score set as the dependent variable, the positive slope for the linear trend line was steeper with vCS; specifically, the values were 2.0 × 10^−8^ and 7.0 × 10^−8^ for single-contact stimulation and vCS, respectively (Fig. 2). Further, the mean of the slopes extracted from the linear trend line was significantly greater with vCS (*P* = 0.031) (Fig. 3).

**Table 1:** UPDRS motor scores of the pre-assessment performed pre-implantation of the DBS system.

Participant	Pre-DBS, OFF L-DOPA UPDRS score	Pre-DBS, ON L-DOPA UPDRS score	Response to L-DOPA (% Change)
1	16	8	50
2	36	22	39
3	20	13	35
4	45	21	53
5	31	15	51
6	31	6	81
7	52	38	27
Average	33.0	17.6	48.1

UPDRS motor scores listed for each participant in the OFF and ON L-DOPA state. Pre-assessments were performed at least one week before surgical implantation of the DBS system. The OFF L-DOPA state required discontinuation of L-DOPA for at least 12 hours and the ON L-DOPA state was reached by patients taking a suprathreshold dose compared to their regular morning dose (typically 130%). The response to L-DOPA (%) was calculated with equation (1): $Levodopa\,response=(\frac{OFF\,levodopa\,score-ON\,levodopa\,score}{OFF\,levodopa\,score\,})\times 100$. Averages were rounded to one decimal place.

**Table 2:** UPDRS motor scores for every investigational setting across the various amplitude stages.

Participant	Setting 1	Setting 2	Setting 3	Setting 4	Setting 5	Setting 6	Setting 7	Setting 8
**Amplitude Stage 1**								
1	8	7	13	5	10	5	6	8
2	16	12	23	18	12	8	22	19
3	29	30	28	28	31	28	26	27
4	24	19	21	17	24	27	24	22
5	31	27	26	28	26	27	30	32
6	47	47	50	48	43	47	48	49
7	31	49	54	36	44	51	52	51
**Amplitude Stage 2**								
1	7	13	11	14	9	10	10	15
2	20	19	26	22	21	20	27	21
3	25	25	26	25	25	21	29	27
4	30	14	18	19	19	28	37	33
5	24	28	28	28	29	26	28	27
6	49	43	42	46	44	43	45	46
7	33	36	35	41	38	32	32	32
**Amplitude Stage 3**								
1	19	17	24	17	21	15	21	15
2	22	17	36	27	17	22	25	23
3	21	15	22	24	18	19	23	21
4	31	19	13	34	17	30	20	20
5	29	26	23	30	20	27	27	27
6	44	40	*	41	40	48	46	41
7	31	35	33	34	36	36	36	38
**Amplitude Stage 4**								
1	19	12	21	22	18	13	21	15
2	40	25	55	29	37	32	21	25
3	22	24	*	22	16	13	22	19
4	20	20	15	21	28	23	23	22
5	31	33	32	28	30	31	33	26
6	50	45	*	45	45	41	42	40
7	29	36	34	33	35	35	32	35

UPDRS motor scores listed for each participant for every investigational setting assessed across the four amplitude stages. The amplitude of the stages increased by adding 20% more of the TW to the TW minimum in a sequential manner; Amplitude Stage 1 occurred around six weeks following surgical implantation. An * denotes an incomplete assessment due to disabling, intolerable dyskinesia or dystonia.

**Table 3:** List of all investigational amplitudes for each participant across all amplitude stages.

Participant	Amplitude Stage (mA)							
	1-Left STN	1- Right STN	2- Left STN	2-Right STN	3-Left STN	3-Right STN	4-Left STN	4-Right STN
1	2.8	1.8	3.6	2.8	4.4	3.9	4.8	4.4
2	1.5	1.4	2.0	1.8	2.5	2.2	3.0	2.6
3	1.7	1.8	2.4	2.6	3.1	3.4	3.8	4.2
4	1.0	1.1	1.5	1.7	2.0	2.3	2.5	2.9
5	1.9	1.6	2.3	2.2	2.7	2.8	3.1	3.4
6	1.9	2.3	2.2	3.1	2.6	3.9	3.0	4.7
7	2.0	1.8	2.5	2.1	3.0	2.4	3.5	2.7
Average	1.8	1.7	2.4	2.3	2.9	3.0	3.4	3.6

All investigational amplitudes for the left and right pair of contacts that were administered and subject to fractionation based on the investigational setting across all amplitude stages. The amplitude of the stages increased by adding 20% more of the TW to the TW minimum in a sequential manner. Averages were rounded to one decimal place.

## Discussion

No differences in the UPDRS motor symptom scores between vCS and single-contact stimulation across stimulation amplitudes were found, which was expected as all administered amplitudes were within the participants’ therapeutic window (TW). Moreover, TEED was minimized when vCS was employed but no differential effect was found on the FAP score when comparing the stimulation types (Fig. 1). Since no significant differences were found between FAP scores in both conditions; it cannot be concluded that vCS improves ambulation to a greater degree (Fig. 1b). Correspondingly, no differences were found in UPDRS motor symptom scores between vCS and single-contact stimulation. However, severe dyskinesia and dystonia that interfered with gait and UPDRS assessments manifested at higher stimulation amplitudes (Amplitude Stage 3 and 4) for Participants 3 and 6 with single-contact stimulation, particularly Setting 3. The variable outcome ranging from symptom alleviation to disabling side effects elicited with single-contact stimulation supports the need for vCS. vCS is not subject to the same variability of the clinical outcome across patients compared to single-contact stimulation, which is beneficial for clinical practice. Further, dyskinesia and dystonia resulting from single-contact stimulation with constant-current within the patients’ TW particularly demonstrates the necessity of vCS to fine-tune current spread through current division. The induction of side effects and poor motor performance with single-contact stimulation is likely attributed to the greater current spread due to a broader charge distribution between the anode and cathode^10^. Notably, vCS has been demonstrated to increase the side effect threshold, allowing for stimulation at higher amplitudes without inducing side effects^28^. Thus, the higher side effect limit of vCS represents greater practicality since one consistently used strategy to address worsened symptom severity associated with disease progression is to increase the stimulation amplitude^30^.

Furthermore, the TEED by vCS was significantly lower than the average TEED by single-contact stimulation (Fig. 1a). vCS minimizes the TEED to an individual contact through current division; thus, the TEED and charge delivery is divided between multiple active contacts rather than involving a single-contact. Lower TEED in vCS reduces power consumption, which prolongs battery life and minimizes side effects by reducing current spread into non-intended neural structures^10,22^. TEED is directly related to the drainage rate of the IPG battery; therefore, reduced TEED can help delay the need for surgical IPG re-implantation to replace the battery. Compared to the initial implantation, re-implantation has been reported to elicit a higher infection rate and often requires a greater frequency of programming changes to achieve symptom alleviation^11,12^. Allert and colleagues reported that 20% of patients experience sub-optimal symptom relief despite accurate hardware restoration and programming with parameters that provided alleviation before IPG replacement^11^. Further, TEED reduction associated with vCS suggests that it is not associated with the same concerns of battery drain as other novel techniques such as interleaving stimulation, which independently and alternately stimulates from two contacts with different voltages and pulse widths at the same frequency^31^. Similar to vCS, interleaving stimulation may be implemented when unipolar and bipolar configurations fail to improve symptoms^31^.

The FAP and TEED values with vCS and single-contact stimulation exhibited a positive correlation; accordingly, FAP scores were directly related to TEED values (Fig. 2). Thus, gait performance improved as the current amplitude increased within the participants’ TW. In general, the enhanced therapeutic effect of increasing the stimulation amplitude within the TW, in both conditions, may act synonymously to a higher dosage of oral pharmacotherapy within the patients’ tolerance. However, the Spearman’s rank correlation coefficient was less positive with single-contact stimulation, which may be attributed to induction of dyskinesia and dystonia at higher stimulation amplitudes. Therefore, the clinical outcome with single-contact stimulation was more variable and sensitive to increases in the stimulation amplitude and spread of the stimulation area^13^. Single-contact stimulation is associated with a greater radial spread that arises from a single part of the electrode; thus, at higher amplitudes a broader current distribution may spread into non-intended structures, which elicits side effects. Conversely, radial current spread is reduced with vCS since the electric field is more elongated as a result of current division between multiple contacts^10^. Accordingly, algorithms relating the current divisions at respective amplitudes to contact localizations may improve the efficacy of neuroanatomical targeting and efficiency of conventional programming, which is ad-hoc and laborious^13^. Moreover, the positive slope for the linear trend line between FAP and TEED was steeper with vCS and the mean of the slopes of each participant’s regression in the vCS stimulation condition was significantly greater (Figs. 2 & 3). A steeper positive slope suggests that vCS elicits higher FAP scores at lower TEED values, which is associated with better gait performance and reduced power consumption and side effect risk, respectively.

Accordingly, these findings support the utility of vCS as it elicits a practical benefit over single-contact stimulation by increasing the side-effect limit, improving gait at lower amplitudes (reduction of the efficacy threshold), and lowering the TEED (reduction of power consumption). Thus, vCS widens the TW, which was demonstrated in the first case study of vCS; symptom alleviation without side-effect induction was elicited at the same amplitude that single-contact stimulation elicited dyskinesia with sub-optimal symptom alleviation^28^. Thus, among patients with L-DOPA responsive gait, vCS may be implemented to improve ambulation with lower TEED values, which reduces the manifestation of side effects and delays the need for IPG replacement due to decreased power consumption and a decreased drainage rate of the IPG battery.

The primary limitation to this investigation was the integrated analysis of segmented and non-segmented contacts, which was performed due to the small sample. TEED values for segmented contacts were inherently smaller because current is divided equally between segments. Thus, segmented contacts stimulate with a lower current and exhibit a lower TEED because the current is squared in the TEED equation (equation 3). Future studies should separate segmented and non-segmented contacts; notably, this may require a substantial increase in the sample size since segmented electrodes are only segmented at two levels. Thus, it is common for patients with segmented electrodes to be stimulated with a combination of segmented and non-segmented contacts. Additionally, the present investigation modelled conventional stimulation with a CC device to facilitate a within-subjects design that was employed due to the small sample and heterogeneous disease phenotype of PD. Nevertheless, a valuable future investigation should compare vCS to VC, single-contact stimulation to more accurately represent conventional practices. Moreover, TEED values may not precisely reflect the administration of a setting since impedance measures were obtained from a single time point, at least nine weeks following implantation, and the literature suggests that impedance may decrease during stimulation^25^. Further, the findings are limited to acute DBS effects as the IPG was turned on and vCS investigations began approximately four- and six-weeks following implantation, respectively. During this period, there is a greater frequency of impedance fluctuations and dosing changes of anti-Parkinsonian medication^25^. Oedema is postulated to increase the electrode impedance while stimulation rapidly decreases the electrode impedance^25^. VC systems are particularly sensitive to impedance fluctuations since they deliver voltage that is directly proportional to the electrode impedance^25,30^. Therefore, investigations during the early post-operative period are valid because this is when vCS is hypothesized to be particularly favourable due to the devices’ ability to address impedance fluctuations by dynamically manipulating the voltage to ensure constant-current delivery and to divide current to fine-tune the shape of the stimulation^13,23^. However, potential confounding effects from dosing changes of medication taken outside of testing sessions must be considered. Accordingly, another limitation would be the inability to completely restrict participants to DBS therapy over the investigational weeks, particularly at the lower stimulation amplitudes, due to medical beneficence. Thus, observed results cannot be fully attributed to DBS despite the supervising Movement Disorders specialist titrating oral medication doses to consistently minimize confounding and the discontinuation of medication for at least 12 hours before testing^32^. Similarly, the washout period of 25 minutes may not be adequate as Cooper *et al*. 2013 found that an adequate washout period for STN-DBS varies across participants and is inversely related with disease duration^33^. Lastly, unequal divisions of 70%/30% modelled vCS but the counterpart (30%/70%) was not investigated; therefore, the more ventrally located contact was biased with a greater proportion of current. Accordingly, future studies should personalize current divisions to optimally address each patients’ symptoms in a larger sample.

## Methods

### Study participants

This study was approved by the Human Subjects Research Ethics Board (REB) at Western University (REB #108453); accordingly, the study was performed in accordance with ethical standards established in the 1964 Declaration of Helsinki and its later amendments. Inclusion criteria included: 1) diagnosis of idiopathic PD with L-DOPA responsive motor symptoms as assessed with the standard L-DOPA challenge test (at least 25%); equation (1): $Levodopa\,response=(\frac{OFF\,levodopa\,score-ON\,levodopa\,score}{OFF\,levodopa\,score\,})\times 100$, 2) disabling motor fluctuations and dyskinesia, and 3) absence of dementia or psychiatric abnormalities confirmed by neuropsychological testing^32^. An adequate motor symptom response to L-DOPA was confirmed at least one week prior to implantation of the DBS system during the pre-assessment. Exclusion criteria included: 1) history of brain surgery, 2) previous implantation of a cardiac pacemaker, 3) overall poor health, and 4) tendency to exhibit lack of compliance. Ultimately, 10 individuals provided informed, written consent prior to their inclusion in the study. However, one patient voluntarily withdrew due to adverse health concerns unrelated to the study, one patient was implanted with a DBS system unable to perform vCS, and one patient had an inadequate motor symptom response to L-DOPA. Overall, seven participants with PD were included in the analysis of this study.

### Surgical implantation and contact selection

All DBS devices (two electrodes and IPG) were implanted on the same day by functional neurosurgeons at University Hospital, London Health Sciences Centre (London, ON, CA). Participants were implanted with the Vercise^TM^ PC IPG and the Cartesia^TM^ Directional electrodes or the Vercise^TM^ IPG and associated electrodes (Boston Scientific, Valencia, CA, USA). Local and general anaesthesia were utilized for electrode and IPG implantation, respectively. The dorsolateral STN was targeted with standard stereotactic coordinates—4.0 millimetres (mm) ventral, 2.0 mm posterior, and 12.0 mm lateral to the mid-commissural point^34^. Intraoperatively, a stereotactic Leksell frame mounted the head for computed tomography imaging and electrode implantation. Spike recordings confirmed the STN positioning in the dorsal to ventral axis from five microelectrodes temporarily implanted to create a central, anterior, posterior, medial, and lateral channel. The implantation trajectory of the therapeutic macroelectrode was determined by the microelectrode that exhibited the most extensive STN spike signature and elicited the greatest improvement of tremor and rigidity upon intraoperative stimulation.

The first TW review was performed one-month post-implantation and involved a unipolar assessment of all contacts to identify the contact in the left and right STN that provided the most extensive TW. The TW was defined by the maximum and minimum or the side effect and efficacy threshold, respectively. The former is defined as the highest amplitude that elicits symptom improvement just before side effect manifestation and the latter is the lowest amplitude that provides symptom improvement. The TW was determined by increasing the current by increments of 0.5 mA until side effect manifestation. Subsequently, the previous amplitude was programmed and increments of 0.1 mA confirmed the exact side effect threshold. Finger taps, upper limb rigidity, and rest tremor were assessed for improvement, and postural tremor was additionally assessed for some participants. Blurry vision, face pulling, dystonia, dysarthria, and any other uncomfortable, intolerable sensations were noted as side effects. The second TW review was performed two weeks later, which paired the initially identified contact with all other contacts to find the pair that elicited the widest TW. Thus, the pair of investigational contacts were not necessarily adjacent and were only selected based on the extent of the TW. During the second review, each contact of the pair was stimulated with 50% of the current. Notably, the IPG was permanently turned on following the initial review, and participants accustomed to stimulation of 0.5 mA outside testing sessions. The frequency (130 Hz) and pulse width (60 microseconds (μs)) were consistent throughout the entire investigation; however, one participant was stimulated with a pulse width of 90 μs because 60 μs was inadequate for symptom alleviation.

### Study design

vCS investigations began immediately after the second contact review, which was approximately six weeks following surgical implantation. vCS was modelled with current divisions of 50%/50% and 70%/30% between two contacts and compared to single-contact stimulation, which amounted to eight investigational settings (Table 4). All settings were implemented with a unipolar configuration; thus, both contacts were set as cathodes for vCS. Directional electrodes were always stimulated in the ring mode; therefore, all three segments were active and current was divided equally. All settings were repeated over four amplitude stages with increasing amplitudes within the TW. The amplitude of Stage 1 was calculated by the sum of the TW minimum and 20% of the TW; equation (2): $Amplitude\,of\,Stage\,1=(TW\,minimum)+[(TW\,maximum-TW\,minimum)\times 0.2]$. For each subsequent stage, amplitude was increased by adding 20% more of the TW to the TW minimum (Table 5). For instance, the stimulation amplitude of Stage 4 added 40% of the TW to the TW minimum. Namely, the TW was calculated by the difference between the TW maximum and TW minimum. Settings were randomized across participants; however, the order remained consistent throughout amplitude stages for each participant. After programming of each investigational setting, a 25-minute washout period was enforced before the subsequent gait assessment.

**Table 4:** Investigational settings of single-contact and vCS stimulation.

Setting Number	Stimulation Model	Left STN	Right STN
1	Single-contact	**A):** 100% **B):** 0%	**A):** 100% **B):** 0%
2	Single-contact	**A):** 100% **B):** 0%	**A):** 0% **B):** 100%
3	Single-contact	**A):** 0% **B):** 100%	**A):** 100% **B):** 0%
4	Single-contact	**A):** 0% **B):** 100%	**A):** 0% **B):** 100%
5	vCS	**A):** 70% **B):** 30%	**A):** 70% **B):** 30%
6	vCS	**A):** 70% **B):** 30%	**A):** 50% **B):** 50%
7	vCS	**A):** 50% **B):** 50%	**A):** 70% **B):** 30%
8	vCS	**A):** 50% **B):** 50%	**A):** 50% **B):** 50%

Summary of the settings used to investigate single-contact and vCS stimulation. **A)** represents the ventral contact and **B)** represents the dorsal contact. vCS was modelled with current divisions of 50%/50% and 70%/30% between two contacts. Investigational settings were randomized across participants but the order remained consistent for each participant as settings were investigated at higher amplitudes.

**Table 5:** Equations to determine the stimulation amplitudes administered at each amplitude stage.

Amplitude Stage	Current Amplitude
1	(TW minimum) + [(TW maximum − TW minimum)×**0.2**]
2	(TW minimum) + [(TW maximum – TW minimum)×**0.4**]
3	(TW minimum) + [(TW maximum – TW minimum)×**0.6**]
4	(TW minimum) + [(TW maximum – TW minimum)×**0.8**]

Settings were investigated at higher amplitudes throughout the sequential amplitude stages by adding 20% more of the TW to the TW minimum. Namely, the TW was calculated by subtracting the TW minimum from the TW maximum.

### Motor evaluations with UPDRS motor scores and gait assessments

The UPDRS assessed motor symptoms at every setting across all amplitude stages as supplementary clinical data as the UPDRS is the gold standard for assessing PD symptom severity. Thus, scores were used to determine if a setting was representative of a PD patient receiving STN-DBS within their TW. UPDRS scores did not include toe tapping, kinetic tremor of the hands, and constancy of rest tremor, which are part of the Movement Disorder Society (MDS)-UPDRS, Part III (motor examination); nevertheless, the rater was certified to administer the *MDS-UPDRS, Part III (motor examination)*. Of note, symptom severity is directly related to the UPDRS score; thus, higher scores indicate greater symptom severity. Moreover, gait assessments were recorded using a 0.61 metre by 6.1 metre Zeno^TM^ walkway embedded with pressure-sensitive sensors (Havertown, PA). Forward walking was assessed at a self-selected pace without shoes, assistive walking devices (verbal cues, splints, or orthoses), or ambulatory aids (canes, crutches, or walkers)^35^. Participants arose from a seated position completing five continuous loops in a clockwise direction to allow for speed maintenance and recordings in a uniform direction—away from the starting position. Overall ambulation was assessed with the FAP score, which was extracted from the ProtoKinetics Movement Analysis Software (Havertown, PA). Gouelle (2014) summarized the calculation of the FAP score as the deduction of points from a maximal score of 100 for aberrations from a healthy gait such as abnormal step time and length, stride velocity, and base of support^35^. Notably, scores between 95 to 100 represent gait in a non-diseased adult population^36^. The UPDRS and gait assessments on the pressure sensitive walkway were administered during the pre-assessment and vCS sessions.

### Dosing protocol of anti-Parkinsonian medication

Participants were permitted to take L-DOPA, amantadine (to treat dyskinesia), and dopamine agonists outside testing sessions if medications were discontinued for at least 12 hours prior to the initiation of the pre-assessment, programming sessions to determine the TW, and investigational setting testing sessions. Thus, anti-parkinsonian medication was never taken during testing sessions with exception of the “ON” assessment of the pre-assessment. Discontinuation for 12 hours was based on the standard for acute challenges, which allows patients to be tolerably functional with adequate drug elimination^32^. The pre-assessment also followed the standard protocol; “OFF” assessments preceded ON assessments during which, participants took a suprathreshold dose compared to their regular morning dose (typically 130%)^32^. As the stimulation amplitude increased throughout the study, dose reductions for medication taken outside of testing hours were prescribed by the supervising clinician to titrate concurrent anti-Parkinsonian therapies. Of note, dopamine agonists were slowly tapered based on the clinician’s discretion to avoid dopamine agonist withdrawal^37^.

### Extraction and calculation of the TEED value

TEED values were calculated for each contact for every setting among the four investigated amplitudes for each participant (Table 5). Voltage was replaced with current according to Ohm’s law and all TEED calculations followed equation (3): $$TEED={(current)}^{2}\times impedance\times $$$$frequency\times pulse\,width$$. Units were as follows: mA, ohms (Ω), Hz, and μs for current, impedance, frequency, and pulse width, respectively. TEED values for segmented contacts stimulated in the ring mode required summation of individual TEED values. TEED calculations for segmented contacts denoted as a, b, and c followed equation (4): $$TEE{D}_{{\boldsymbol{abc}}}=TEE{D}_{contact{\boldsymbol{a}}}+TEE{D}_{contact{\boldsymbol{b}}}+TEE{D}_{contact{\boldsymbol{c}}}$$. Accordingly, setting TEED values were calculated by summating TEED values for both contacts of the left and right electrode, following equation (5): $$Setting\,TEED=TEE{D}_{LeftContactA}+TEE{D}_{LeftContactB}+TEE{D}_{RightContactA}+TEE{D}_{RightContactB}$$. Calculations incorporated the respective current division of each setting; for instance, a division of 50%/50% resulted in an equal separation of current amplitude between two contacts. Additionally, current amplitudes were divided equally among segmented contacts; therefore, 50% of the amplitude was divided by three for each segment. Impedance values between the IPG and each individual contact or segment were used since a unipolar electrode geometry was employed. Impedance values were retrieved from one time-point at least nine weeks after surgical implantation to allow for consistency.

### Data and statistical analyses

Statistical analyses were performed by GraphPad Prism 6.00 (La Jolla, CA, USA) and R (version 3.5.1, Boston, MA, USA) using an alpha criterion of ≤0.05. Analyses utilized GraphPad Prism unless specified. Outliers for UPDRS and FAP scores were identified through diagnosis of disabling dyskinesia or dystonia by the supervising clinician. FAP scores were subject to additional outlier identification through the interquartile range rule; the FAP score and associated TEED value were removed if the FAP score was below the difference of the first quartile and the interquartile range multiplied by 1.5 or above the sum of the third quartile and the interquartile range multiplied by 1.5. Of note, UPDRS scores did not undergo outlier identification with the interquartile range rule because it served as supplementary clinical data. All data sets had a sample of seven (N= 7) and normality was assessed using the Shapiro-Wilk test. Differences in UPDRS scores between the two stimulation conditions and across the amplitude stages were identified with the Skillings-Mack test and a Nemenyi post-hoc test was planned; the former and latter were performed using R packages “Skillings.Mack” and “PMCMRplus”, respectively. Namely, the Skillings-Mack test is a non-parametric test used in place of the Friedman test (similar to the parametric repeated measures ANOVA) when there is missing data, and the Nemenyi post-hoc test is a conservative test used to perform pair-wise comparisons^38,39^. The effect of vCS and single-contact stimulation on FAP and TEED independently was assessed using the two-tailed, Wilcoxon matched-pairs signed-rank test. Additionally, a Spearman, two-tailed, correlation was performed to identify the potential correlation between FAP and TEED. Lastly, the regression between FAP and TEED in both conditions was compared by using a two-tailed, Wilcoxon matched-pairs signed-rank test. Differences between means of the slopes of linear trend lines for each participant’s regression were assessed with the FAP score set as the dependent variable.

### Ethical Standards

This study was approved by the Human Subjects Research Ethics Board (REB) at Western University (REB #108453); accordingly, this study has been performed in accordance with the ethical standards established in the 1964 Declaration of Helsinki and its later amendments. Additionally, all study participants provided informed written consent prior to their inclusion in the study.

## Data Availability

The analysed datasets that support the study results are available from the corresponding author upon reasonable request.
